# Characterization of clinical patterns of dengue patients using an unsupervised machine learning approach

**DOI:** 10.1186/s12879-019-4282-y

**Published:** 2019-07-22

**Authors:** Gleicy Macedo Hair, Flávio Fonseca Nobre, Patrícia Brasil

**Affiliations:** 1 Laboratório de Engenharia em Sistemas de Saúde, Programa de Engenharia Biomédica/COPPE/UFRJ, Centro de Tecnologia - Bloco H - Sala H327, Caixa Postal (P.O. Box): 68510, Cidade Universitária, Ilha do Fundão, Rio de Janeiro, RJ 21941-972 Brazil; 2 Acute Febrile Illnesses Laboratory, Evandro Chagas National Institute of Infectious Diseases; Oswaldo Cruz Foundation (Fiocruz), Rio de Janeiro, RJ Brazil

**Keywords:** Dengue, Age, Clinical classification, Warning signs, Machine learning

## Abstract

**Background:**

Despite the greater sensitivity of the new dengue clinical classification proposed by the World Health Organization (WHO) in 2009, there is a need for a better definition of warning signs and clinical progression of dengue cases. Classic statistical methods have been used to evaluate risk criteria in dengue patients, however they usually cannot access the complexity of dengue clinical profiles. We propose the use of machine learning as an alternative tool to identify the possible characteristics that could be used to develop a risk criterion for severity in dengue patients.

**Method:**

In this study, we analyzed the clinical profiles of 523 confirmed dengue cases using self-organizing maps (SOM) and random forest algorithms to identify clusters of patients with similar patterns.

**Results:**

We identified four natural clusters, two with features of dengue without warning signs or mild disease, one that comprises the severe dengue cases and high frequency of warning signs, and another with intermediate characteristics. Age appeared as the key variable for splitting the data into these four clusters although warning signs such as abdominal pain or tenderness, clinical fluid accumulation, mucosal bleeding, lethargy, restlessness, liver enlargement and increased hematocrit associated with a decrease in platelet counts should also be considered to evaluate severity in dengue patients.

**Conclusions:**

These findings suggest that age must be the first characteristic to be considered in places where dengue is hyperendemic. Our results show that warning signs should be closely monitored, mainly in children. Further studies exploring these results in a longitudinal approach may help to understand the full spectrum of dengue clinical manifestations.

## Background

Dengue is an acute and systemic disease caused by the dengue virus (DENV), with a broad clinical spectrum ranging from asymptomatic to severe infections. Most infections by DENV result in a mild disease known as dengue without warning signs, but a small proportion of patients develop the severe form [1].

Although the World Health Organization (WHO) issued the revised dengue guideline with a new clinical classification [2], there is still debate regarding its specificity [3, 4]. This new classification grouped the patients according to the presence or absence of warnings signs and severe dengue. Studies evaluating the new classification demonstrated a greater sensitivity when applied in endemic regions in both prospective and retrospective data, but the authors highlighted the need for a better definition of warning signs, mainly in the absence of laboratory tests [5, 6].

Classic statistical methods have been used to evaluate warning signs and determine risk criteria for severity in dengue patients [7–9], however, the complexity of the clinical profiles and the many overlapping levels of severity makes the disease prognosis nearly impossible to predict. The challenge in modeling this type of clinical data in a multifactorial disease such as dengue relies on the choice of an appropriate modeling approach that instead of providing a single predictive attribute, considers a combination of variables regardless of the data structure.

The use of unsupervised machine learning techniques is becoming popular in the medical field to reduce the dimensionality of the data and to help visualize possible patterns. The self-organizing map (SOM) is especially suitable for this task because it projects the high dimension data into a low dimension without losing the data structure [10]. Due to the increase in volume and complexity of data over the last decade, especially in the medical field, several variants of the SOM algorithm were introduced to generalize the original algorithm to handle both numerical and categorical attributes. When data are described by categorical variables or by relations between objects, a common solution is to use a measure of resemblance (i.e., a similarity or a dissimilarity measure). A general extension of this idea is a stochastic version of SOM that can be used to analyze dissimilarity data [11].

Another machine learning technique that has been successfully used in the medical field is the random forest [12]. It provides a proximity scores matrix that assesses the number of samples and the similarity/dissimilarity matrices between them. Both matrices (similarity and dissimilarity) can be used to perform a powerful unsupervised analysis to identify patterns in the data structure.

In this study we combined random forest followed by stochastic SOM to visualize possible natural clusters associated to clinical natural patterns in dengue confirmed cases. These clusters were then reviewed for possible characteristics that could be used as risk criteria in dengue patients.

## Methods

### Study population and eligibility criteria

In this descriptive cross-sectional study, we analyzed retrospective data of patients with suspicion of dengue infection, assisted at the hospital of the Instituto Nacional de Infectologia Evandro Chagas/FIOCRUZ, Brazil between January 2007 and December 2013. Following the age shifting in Brazil in 2007, the Instituto Nacional de Infectologia Evandro Chagas started a project to study dengue infection in children in collaboration with three pediatric hospitals in the city. These hospitals also serve as primary care and tertiary care for dengue, therefore patients with suspicion of dengue infection and admitted into these pediatric hospitals in Rio de Janeiro RJ were also included in this analysis.

The inclusion criteria were laboratory-confirmed dengue virus (DENV) cases enrolled up to 7 days from the onset of the symptoms and followed until the outcome (cure or death), which encompassed the acute and critical phases of the disease. Patients with comorbidities and cases with more than 7 days after the onset of the symptoms at the time of admission were excluded from this analysis. Only subjects with complete data for all variables including laboratorial and clinical dengue classification were included in our analysis. All dengue cases included in this study were confirmed by at least two of the following criteria: (i) positive DENV-specific real-time reverse transcription polymerase chain reaction (RT-PCR) for any serotype (I-IV) (QIAamp Viral RNA Mini Kit, Qiagen, Hilden, Germany, following the protocol described in Lanciotti et al. [13]), (ii) at least one positive DENV-specific immunoglobulin M (IgM) antibody in the convalescent serum compared to in the acute-phase serum or positive for qualitative IgM with dengue clinical profile during epidemic periods. Tests for detection of anti-dengue IgM were conducted using an antibody-capture enzyme-linked immunosorbent assay (PanBio, Brisbane, Australia), and/or (iii) NS1 antigen capture by using the Platelia™ Dengue NS1 Ag-ELISA Kit (Bio-Rad Laboratories, Marnes-La-Coquette, France) in the acute-phase serum (up to 3 days after the onset of fever), (iv) clinical-epidemiological diagnosis during epidemic periods .

### Data preprocessing and clinical classification

Data were obtained from each patient’s medical records. We created a categorical variable called “age group” (≤ 18 years old and >  18 years old) to better define the distribution of the variables among children and adults in the exploratory analysis. The variables age and days were normalized to reduce its variability before using to define the similarity matrix. Variables such as hematocrit and platelet increase/decrease as well as imaging data to define cavities fluid accumulation were based on at least two tests of complete blood count analyses and X-ray images, respectively. These variables were used to define some warning signs, but they were not included in this analysis.

The clinical classification was performed by trained clinicians based on the WHO guideline. It was then used to compare to the natural clusters defined by the unsupervised neural network. The classification divided the patients into three groups: dengue without warning signs, dengue with warning signs and severe dengue. Warning signs included: abdominal pain or tenderness; persistent vomiting (more than 5 times in 6 h or more than 3 times in 1 h); clinical fluid accumulation including pleural effusion and ascites identified as a reduction of vesicular murmur or reduction of thoracic-vocal trill; abdominal distention or dullness decubitus, confirmed by abnormal imaging findings; mucosal hemorrhage (gastrointestinal hemorrhage and/or metrorrhagia); lethargy (alteration of consciousness and/or Glasgow score < 15) or irritability; and liver enlargement (> 2 cm below the costal margin). Laboratory findings were defined as follows: thrombocytopenia (platelet count, 50,000/mm^3^) and hematocrit change of 20%, either raised or decreased by 20% from the baseline value during the convalescent period. Severe dengue was defined by the following characteristics: (i) Plasma leakage resulting in shock or fluid accumulation with respiratory distress. Shock was defined as the presence of at least 2 of the clinical signs of hypoperfusion, with or without an associated weak pulse pressure (≤20 mmHg) or hypotension for the specified age (decrease in blood arterial systolic pressure 5th percentile for age [<PAS5], calculated as age [years] 2 + 70) [14]; or (ii) severe bleeding, or (iii) severe organ involvement, e.g., severe hepatitis (aspartate aminotransferase/alanine aminotransferase levels > 1000 IU/L); Multiple-organ dysfunction syndrome was considered when dysfunction involved 2 or more organs. Definition and clinical criteria of these signs and symptoms are better described elsewhere [15, 16].

### Unsupervised machine learning techniques

#### Random forest

Random forest is an algorithm based on constructing a binary tree using recursive partitioning. Each binary split recursively divides the parent branch into homogeneous or near homogeneous daughter nodes (the ends of the tree). The trees are built using a two-stage randomization procedure. The first stage introduces the randomization using a bootstrap sample of the original data, and in the second, the randomization is introduced at the node level, by selecting a random subset of variables, and only those variables that keep the purity (homogeneity) of the node are kept during the split. This homogeneity is calculated by the Gini Impurity Index (Eq. 1) and it determines the purity of each node based on the relative frequency of the class in the node being evaluated.

1 $$ G(S)=1-\Sigma {\pi}_i^2 $$

where S is the node being evaluated and π_i_ is the frequency of the class k in the node S.

The advantage of this two-step randomization relies on the generation of decorrelate trees besides the guarantee that even those weak features are considered in the analysis [12]. An “out-of-bag” (OOB) error rate for each observation is calculated using the samples not included in the bootstrap and it is determined by majority vote across trees. Each tree is unpruned to obtain low-bias trees while bagging and random variable selection results in low correlation of the individual trees. Thus, the algorithm yields an ensemble that can achieve low bias and low variance [12].

Random forests can be used as an unsupervised technique. This approach involves the generation of a synthetic dataset to represent data without dependence. The synthetic dataset is appended to the original one, and a two-level classification variable (“original” and “synthetic”) is created. Then a supervised random forest predictor is constructed to classify original from synthetic data [17]. One important output information provided by random forest predictor is a measure of the internal structure of the data (the proximity between data points). This proximity can be determined by examining the node membership of the data. Once this process is done for all trees, the proximities are normalized by dividing them by the total number of t-trees. These scores are then stored in a proximity matrix. This matrix can be used to calculate a dissimilarity matrix by subtracting one from each of the elements, allowing a direct comparison for clustering and visualization approaches to detect data structures in high-dimensional space [17].

The Random forest algorithm was applied in an unsupervised setting to calculate the dissimilarity matrix (SOM input). All analyses were performed by using randomForest package in R [18].

#### Stochastic self-organizing maps (SOM)

SOM is a neural network that uses unsupervised competitive learning to map nonlinear statistical relationships between high-dimensional data into low dimensional grids while maintaining its original topology. Its architecture usually consists of a two-dimensional grid (input and output) with each cell in the array having a processing unit called “neuron”. The neurons are connected to adjacent ones by a neighborhood function and the data points closest to each other in the input space are mapped into nearby neurons on the grid [19].

The SOM training is iterative, and it uses competitive learning where the neurons of the output layer compete to be updated. First, there is a competitive learning phase where a sample *xi* is randomly chosen from the input data and the distance (Euclidean Distance) between the sample and all prototypes *p* are computed [19].

The closest neuron to the input is declared the winning neuron or Best Matching Unit (BMU) p_*u*_, _*u* ϵ {1, ...,U}_, and it can be calculated by Eq. 2.

2 $$ f\left({x}_i\right)=\arg {\min}_{u=1,\dots, U}\left\Vert {x}_i-{p}_u\right\Vert $$

where ||.|| is the Euclidean distance in *ℜ*^*d*^.

The next step consists of a cooperative phase identifying BMU’s neighboring neurons using a neighborhood Gaussian function. Finally, all the prototype vectors are updated. It can be performed either by updating all prototypes via a weighted average (batch SOM) or in a stochastic version where the prototypes are updated mimicking a stochastic gradient descent scheme as described by the Eq. 3. The resulting grid shows the relationship between the neurons displaying the distances between input data [19].

3 $$ \forall u=1,\dots, U\to {p}_u\left(t+1\right)={p}_u(t)+\alpha .h\left(d\left(f\left({x}_i\right)u\right)\right)\left(x-{p}_u(t)\right) $$

where *t* means time, α(*t*) learning rate and *h* is a neighborhood kernel function centered on the winner unit.

The best grid is usually chosen by checking two error measures: (1) quantization error and (2) topographic error. The topographic error or *Te* (Eq. 4) quantifies the continuity of the map with respect to the input space metric by counting the number of times the second-best matching (BMU2) unit of a given observation belongs to the direct neighborhood of the BMU for this observation, whereas the quantization error or *Qe* (Eq. 5) provides the mean distance between each vector and the cluster prototypes for *k* clusters [10].

4 $$ Te=\frac{1}{n}\sum \limits_{k=1}^nu\left({x}_k\right) $$

where *u(x*_*k*_*)* is 1 if the winning neuron (BMU1) and the second neuron (BMU2) are neighbors and 0 if they are not neighbors.

5 $$ Qe=\frac{\sum_{k=1}^n\left\Vert {x}_k-{\omega}_{BMU}\right\Vert }{n} $$

where the mean error corresponding to the difference between the input vector (*x*_*k*_) and vector weight (ωBMU).

**Fig. 1:**
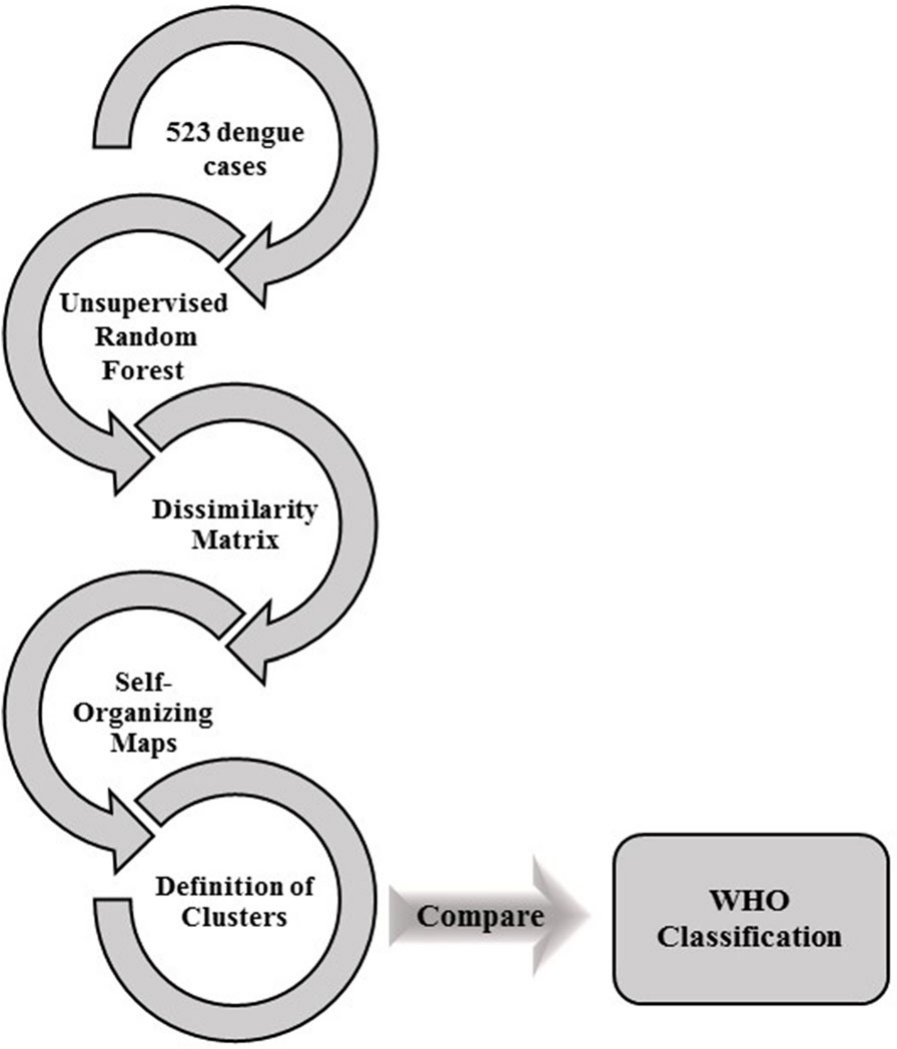
Workflow

Several variants of the SOM algorithm have been introduced to overcome its limitations such as the inability of distinct types of variables and/or structure [11, 20, 21]. One of the extensions relies on the computation of a measure of similarity or dissimilarity as input data where a natural Euclidean structure is not necessarily existent, instead, the dissimilarity between the observations can be described by a dissimilarity measure ∆, where ∆ = (*δ*_*ij*_)_*i,j*_ = 1,...,n, such that ∆ is non negative (*δ*_*ij*_ ≥ 0), symmetric, (*δ*_*ij*_ = *δ*_*ji*_) and null on the diagonal (δ_ii_ = 0).. In this case, the Eq. 2 cannot be carried out straightforwardly since the distances between the input data and the prototypes are not be directly computable. The solution is based on the pseudo-Euclidean framework which considers the prototypes as symbolic convex combinations of the original data [11]. In the stochastic version, it is calculated as described in the Eqs. 6 and 7 which are modification of the Eqs. 2 and 3.

6 $$ f\left({x}_i\right)=\arg {\min}_{u=1,\dots, U}D{\left({\gamma}_u\right)}_i-\frac{1}{2}{\gamma}_u^TD{\gamma}_u $$

7 $$ \gamma \left(t+1\right)\leftarrow {\gamma}_u(t)+\alpha .H\left(d\left(f\left({x}_i\right),u\right)\right)\left({1}_i-{\gamma}_u(t)\right) $$

where γ_u,_ given *u = 1, …, U* are the convex combinations of the input data and **1**_*i*_ is a vector with a single non-null coefficient at the *i-th* position, equal to one. Here we apply this technique to reduce the high-dimensionality and find natural patterns in the data by using the package SOMbrero in R [22]. This package provides the use of dissimilarity measures as input data and an ascending hierarchical clustering algorithm on the prototypes of the trained grid (superClass) for visualization of the natural clusters. The workflow of the methodologies applied is summarized in the Fig. 1.

### Statistical analysis

For descriptive analyses, frequency and percentages were used for categorical variables. For continuous variables, median, range and IQR were used. Categorical variables were compared by using Chi-square test, whereas Fisher’s exact test was performed when the expected table values were smaller than 5. The difference by age and days after the onset of the symptoms among the natural clusters were compared by a one-way ANOVA analysis. Significant differences considered *p*-value < 0.05. Statistical analyses were performed using the R statistical software R 3.5.1 [23].

## Results

From 710 cases reported as dengue during the studied period, 93 were excluded due the lack of laboratorial data and/or missing information and/or had comorbidity, 48 had more than 7 days after the onset of the symptoms, 46 were not able to be classified due to missing information for one or more clinical variables. It resulted in 523 confirmed dengue cases that were used to identify natural patterns in the clinical profiles of patients up to 7 days of disease based on the presence of 30 different variables used for dengue clinical classification. Overall, the average age was 31 years and 49.1% were female. One hundred sixty-two (31%) were children (≤ 18 years old) and 361 (69%) were adults (> 18 years old). Dengue serotypes were identified in 183 (35%) of the patients. The profile of the 523 patients included in this study and serotypes identified are shown in Table 1.

**Table 1:** Demographic characteristics and serotypes of the studied population according to the clinical classification

	Dengue without warning sing (*n* = 293)	Dengue with warning signs (*n* = 160)	Severe dengue (*n* = 70)
Sex (n, female/male)	(140/153)	(72/88)	(45/25)
Age (years)	Min. 0	Min. 0	Min. 0
	Mean 31	Mean 31	Mean 30
	Max. 79	Max. 82	Max. 67
Age group (n, ≤ 18 years old/> 18 years old)	(72/221)	(61/99)	(29/41)
Dengue serotype			
DENV-1 (%)	20 (6.8)	10 (6.2)	3 (4.3)
DENV-2 (%)	21 (7.2)	7 (4.3)	2 (2.9)
DENV-3 (%)	4 (1.36)	4 (2.5)	1 (1.42)
DENV-4 (%)	79 (27)	23 (14.4)	9 (12.8)

*N* Number of patients

The more frequent clinical signs/symptoms in the children vs. adults were nausea/ vomiting (59.2%), persistent vomiting (56.2%), history of abdominal pain (49.4%), abdominal pain or tenderness (48.8%), petechiae (38.3%), clinical fluid accumulation (pleural effusion/ascites) (31%) and liver enlargement > 2 cm (21.6%). Adults presented more myalgia (79%), retro-orbital eye pain (55.1%), arthralgia (53.4%) than children. The frequency of these signs and symptoms by age group are described in the Table 2.

**Table 2:** Distribution of clinical variables by age group

Variables	Abbreviations	≤18 years old *n* = 162 (%)	> 18 years old *n* = 361 (%)
Nausea/ vomiting	*nau/vom*	96 (59.2)	142 (39.3)
Rash	*rash*	69 (42.6)	177 (49)
History of abdominal pain	*abpainhist*	80 (49.4)	118 (32.7)
Myalgia	*myal*	101 (62.3)	285 (79)
Arthralgia	*athral*	47 (29)	193 (53.4)
Pain behind the eyes (retro-orbital eye pain)	*retpain*	58 (32.1)	199 (55.1)
+ tourniquet test	*tourniq+*	5 (3.1)	6 (1.6)
Petechiae	*pet*	62 (38.3)	96 (26.6)
Leukopenia	*leukop*	82 (50.6)	175 (48.5)
Abdominal pain or tenderness	*abpain.ws+*	79 (48.8)	113 (31.3)
Persistent vomiting	*pvom.ws*	91 (56.2)	124 (34.3)
Clinical fluid accumulation (pleural effusion/ascites)	*cfa.ws*	50 (31)	32 (8.8)
Mucosal bleed	*bleed.ws*	53 (32.7)	79 (21.8)
Lethargy, restlessness	*letha/rest.ws*	16 (9.9)	19 (5.2)
Liver enlargement > 2 cm	*liveren.ws*	35 (21.6)	31 (8.6)
Increase hematocrit + decrease of platelet count	*hto/plt.ws*	40 (24.7)	41 (11.3)
Dehydration	*dehyd.spl* ^a^	37 (22.8)	84 (23.2)
Edema	*edema.spl*	16 (9.9)	23 (6.4)
Hypotension	*hypo.spl*	15 (9.2)	17 (4.7)
Narrow pulse pressure < 20 mmHg,	*pp < 20 mmHg.spl*	5 (3)	6 (1.6)
Cold clammy skin/cyanosis	*coldskin.spl*	8 (5)	9 (2.5)
Rapid and weak pulse	*rwp.spl*	10 (6.1)	11 (3)
Slow capillary filling	*scf.spl*	16 (9.9)	15 (4.1)
Respiratory distress	*respdist.spl*	15 (9.2)	19 (5.2)
Severe bleeding	*severeb*	28 (17.3)	37 (10.2)
Impaired consciousness	*neuro.soi* ^b^	4 (2.4)	18 (5)
Aspartate transaminase/alanine transaminase > = 1000	*alt/ast > 1000.soi*	3 (1.8)	4 (1.1)

+ Warning Sign, ^a^Severe Plasma Leakage, ^b^Severe Organ Involvement

The resulting dissimilarity matrix generated by unsupervised random forest was then used as input to perform the stochastic SOM algorithm. One resulting grid was selected after several trainings based on the final energy and the topographic and quantization errors. The grid with the lowest topographic error (0.05) and quantization error (0.43) was chosen (Table 3). The lowest topographic error provides the best representation of the data structure on the grid; therefore, it was prioritized.

**Table 3:** SOM training features

Metric	Mean of 10 grids	Chosen grid
Topographic Error	0.1220	0.0554
Quantization Error	0.4241	0.4388
Final Energy	0.0120	0.0175

**Fig. 2:**
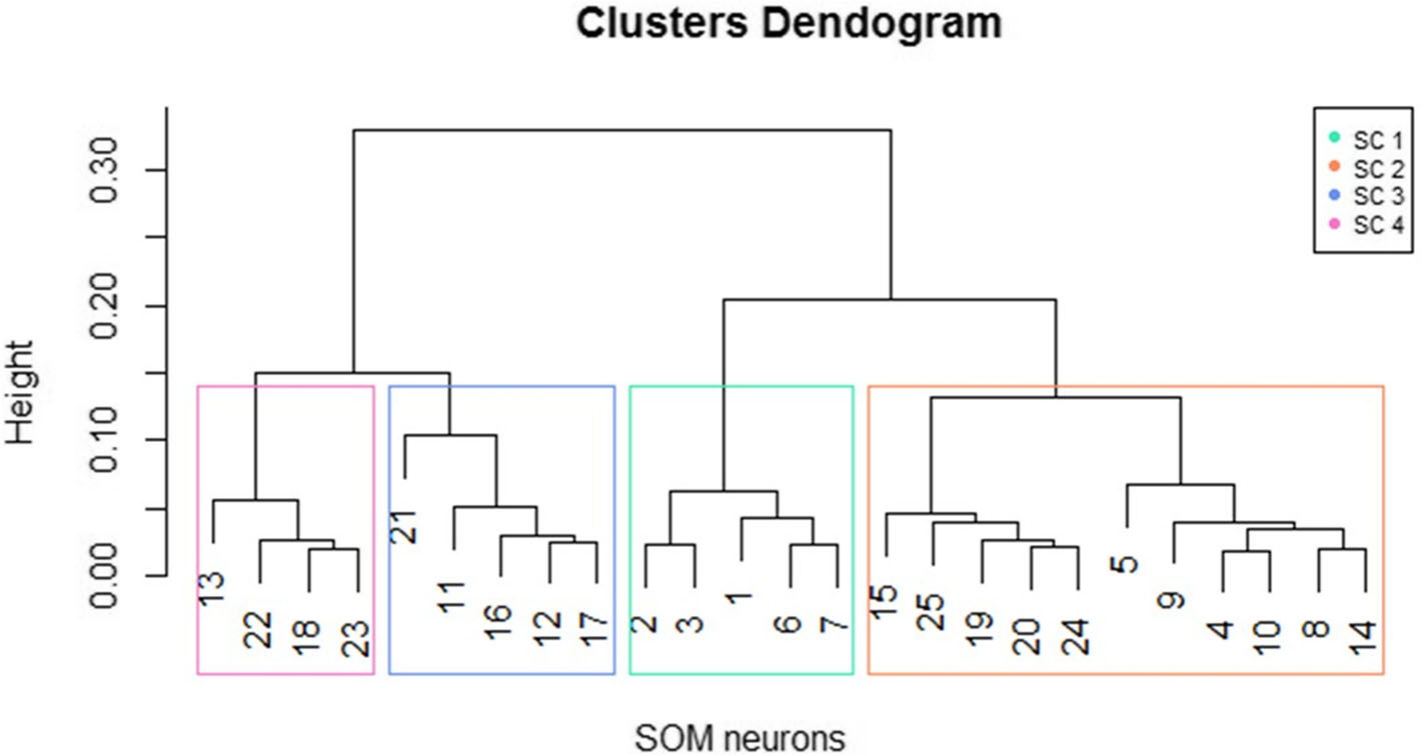
Hierarchical cluster analysis applied on SOM prototypes. SC Super Cluster

A hierarchical clustering was then applied on the SOM prototypes to better understand and visualize the structure in the grid resulting in a dendrogram (Fig. 2), from which 4 clusters were considered as the best division. The best division was selected based on a non-parametric MANOVA described by Anderson [24]. This test is a multivariate analogue to Fisher’s F-ratio and is calculated directly from any symmetric distance or dissimilarity matrix. The *P*-values are then obtained using permutations of the observations to obtain a probability associated with the null hypothesis of no differences among clusters. For 4 clusters, the results were F: 6·57 and *p-value* < 0·001.

The numbers in each node in Fig. 2 correspond to one neuron of the SOM grid and they were grouped according to the clusters defined by the MANOVA analysis (rectangles). These clusters were then all labeled with the patient’s number to obtain the classification and clinical profile from the original data (Table 4).

**Table 4:** Clusters according to the WHO (2009) classification

Classification	Cluster 1	(%)	Cluster 2	(%)	Cluster 3	(%)	Cluster 4	(%)	^a^X-squared	*P*-value
Dengue without WS	48	(78·7)	72	(58)	97	(75·2)	76	(36·3)	64·95	5·11e-14
Dengue with WS	11	(18)	38	(30·7)	29	(22·5)	82	(39·2)	15·88	0·001
Severe Dengue	2	(3·3)	14	(11·3)	3	(2·3)	51	(24·5)	41·33	5·55e-09
Total	61	(100)	124	(100)	129	(100)	209	(100)	–	–

*WS* Warning signs, ^a^Chi-Square test: H0 = all the proportions are equal; H_1_ = At least one proportion is different

**Fig. 3:**
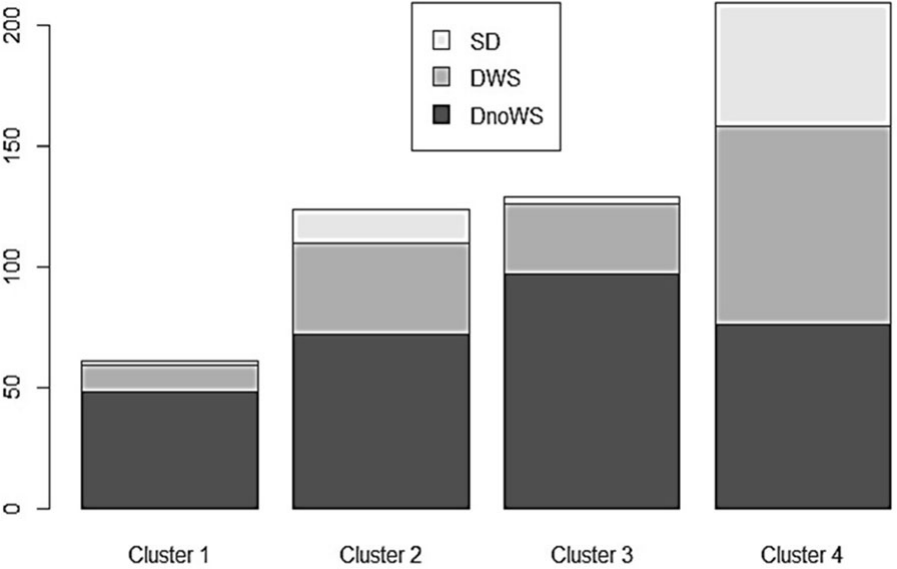
Distribution of the WHO classification by cluster. SD Severe Dengue, DWS Dengue with Warning Signs, DnoWS Dengue without Warning Signs

From 523 patients analyzed, 61 were grouped in cluster 1, 124 in cluster 2, 129 in cluster 3 and 209 in cluster 4. According to the specialist’s classification (Table 4), 78·7% and 75·2% of the patients in clusters 1 and 3 were classified as Dengue without Warning Signs only (*p-value*: 5·11e-14). Clusters 2 and 4 had 30·7% and 39·2% of the patients classified as Dengue with warning signs (*p-value*: 0·001) respectively, however, cluster 4 had the highest percentage of patients classified as severe dengue, which characterize this group as more severe than the others (*p*-value:5·55e-09). The distribution of the WHO classification by clusters is shown in the Fig. 3.

**Fig. 4:**
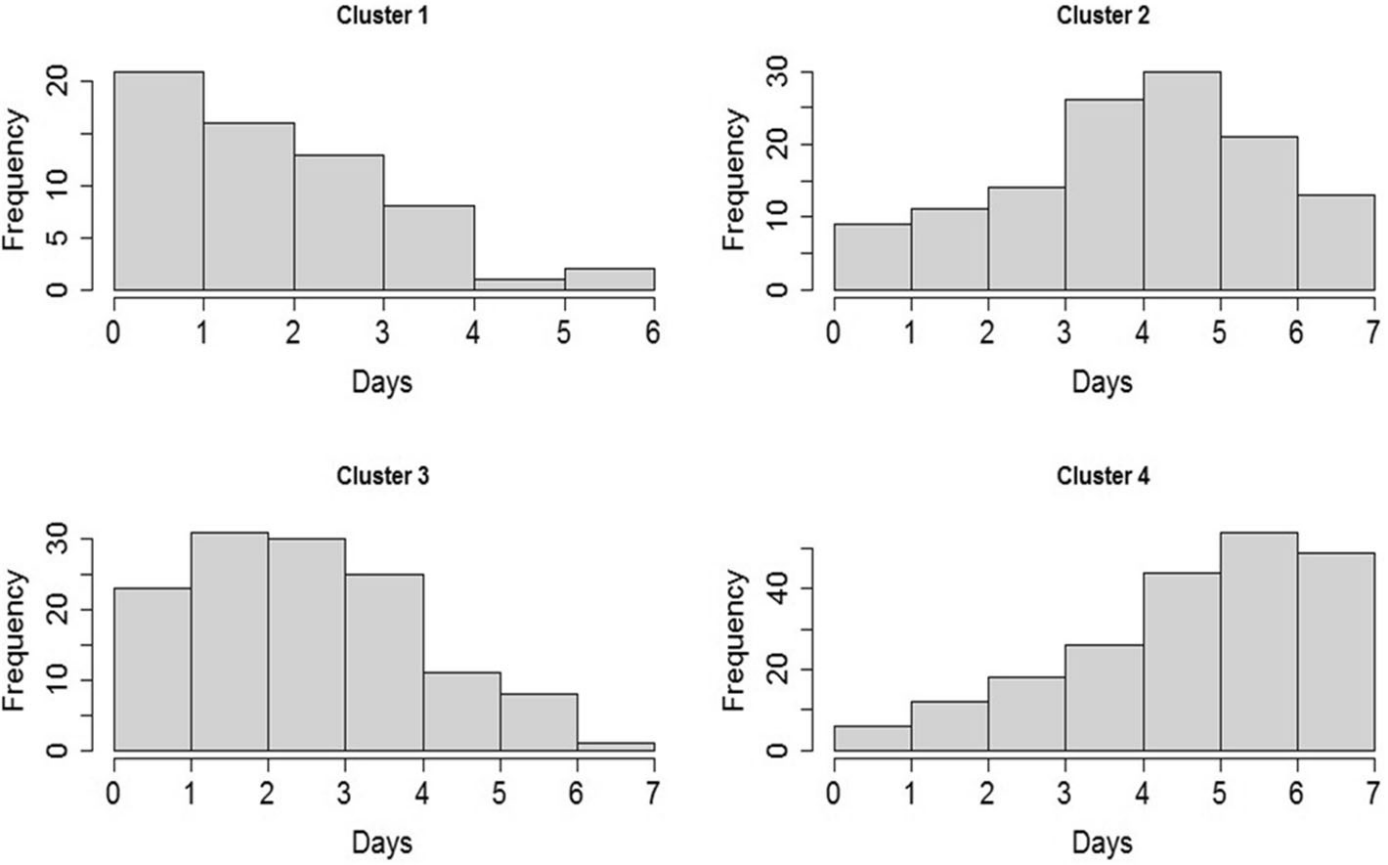
Days after the onset of the symptoms according to the clusters defined by SOM^±^. ± There was a statistical difference between four clusters: *ANOVA F = 14.43, p-value = 4.85e-09*

The distribution of days after the onset of symptoms between clusters was also analyzed (Fig. 4). The patients in cluster 1 were between the 1st and 3rd day of disease. Cluster 3 showed higher frequency of patients between the 2nd and 4th days. Clusters 2 and 4 showed both a higher frequency of patients between 4th - 6th and 5th–7th days of disease respectively (*p-value < 0.05*).

**Fig. 5:**
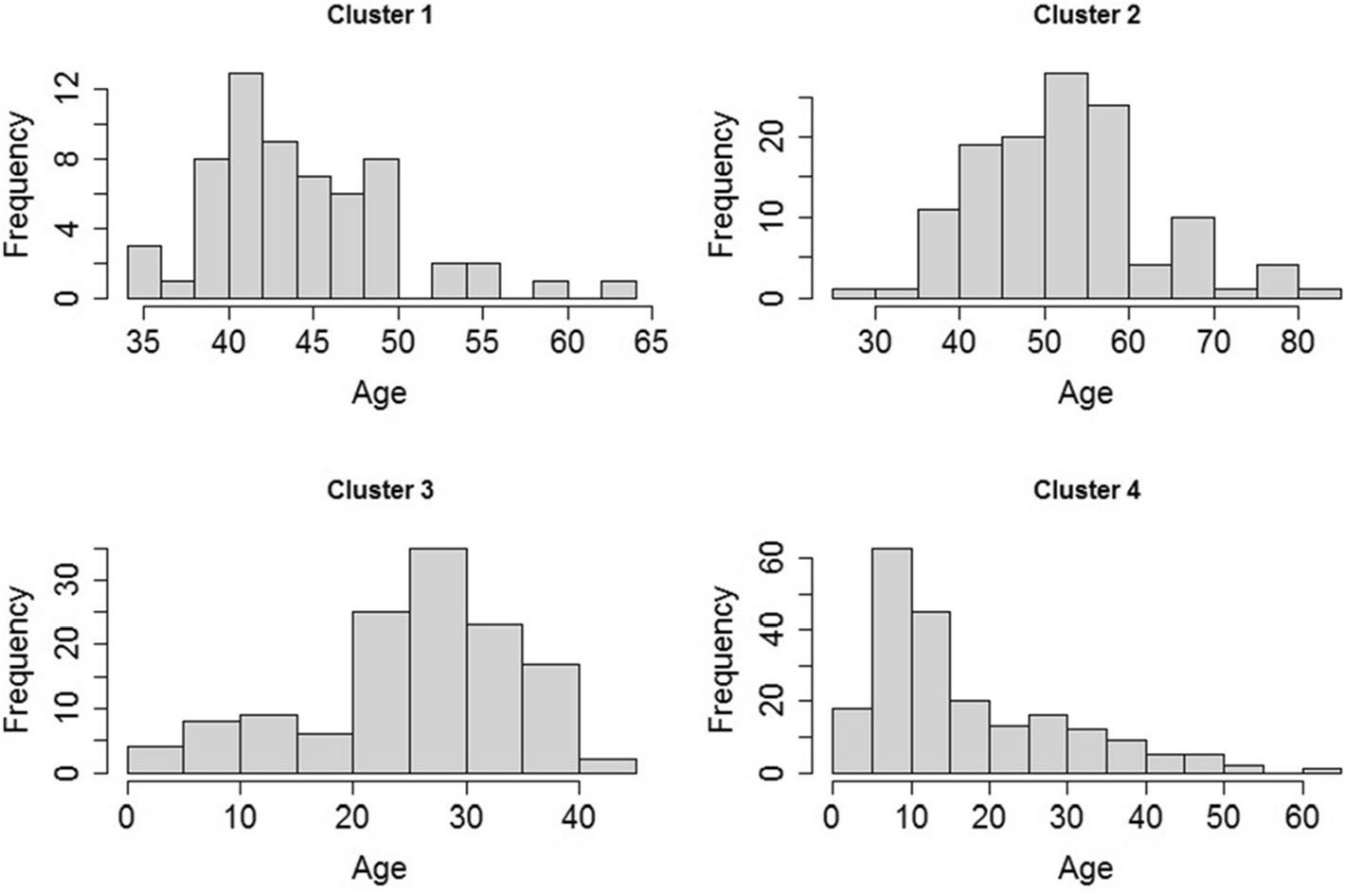
Age distribution according to the clusters defined by SOM^±^. ± There was a statistical difference between four clusters: ANOVA *F* = 338.2, *p*-value = <2e-16

The age distribution between the clusters showed that clusters 1 and 2 concentrate the older patients (age range between 40 and 80 years old), cluster 3 had a higher frequency of young adults (20–40 years old) and cluster 4 consisted mainly of children (5–15 years old) (Fig. 5).

A pairwise analysis was then applied to compare the proportions of each variable between the clusters. The adjusted *p*-values are shown in the Table 5.

**Table 5:** Adjusted^c^ *p*-values of the clinical characteristics according to each pair of clusters

Cluster	1vs3	2vs4	2vs3	2vs1	3vs4	1vs4
nau/vom	0.0001	7.9e-05	0.0001	8.1e-10	< 2e-16	< 2e-16
rash	1	1	1	0.7700	0.7700	0.3500
abpainhist	1	7.4e-08	1	1	1.8e-06	8.1e-06
myal	1	0.0061	0.2200	0.3661	1.6e-06	0.0008
athral	1	5.6e-05	1	0.6600	9.0e-07	5.5e-06
retpain	0.1557	0.0009	0.0004	0.2265	1.7e-14	9.9e-05
tourniq+	1	1	1	1	1	1
pet	0.1980	0.0899	0.0143	0.0027	1.7e-06	3.2e-06
leukop	0.4453	0.0016	0.4453	1	4.4e-07	0.0290
abpain.ws+	0.2800	3.4e-05	0.2800	0.0480	4.6e-09	2.9e-08
pvom.ws	0.0008	5.3e-06	0.0012	1.1e-07	7.6e-16	< 2e-16
cfa.ws	–	1.2e-07	0.0110	0.0730	4.9e-13	4.8e-07
bleed.ws	0.9560	0.0094	0.1367	0.4494	6e-07	0.0013
letha/rest.ws	1	0.3007	0.1439	0.3007	0.0017	0.0401
liveren.ws	1	0.0003	0.0338	0.1060	1.3e-08	8.2e-05
hto/plt.ws	0.7007	0.0005	0.0005	0.0713	3.0e-11	2.8e-05
dehyd.spl^a^	0.0070	0.6579	0.9825	0.0061	0.6579	0.0003
edema.spl	0.5637	0.2934	0.3869	0.3007	0.0074	0.0271
hypo.spl	1	0.4700	0.1440	0.3010	0.0050	0.072
pp < 20 mmHg.spl	–	1	0.4800	0.9700	0.4400	0.9700
coldskin.spl	1	0.0910	1	1	0.0910	0.2660
rwp.spl	1	0.0128	1	1	0.0036	0.0766
scf.spl	1	0.0006	1	1	0.0005	0.0178
respdist.spl	1	0.0400	0.5924	0.5924	0.0008	0.0271
severeb	1	0.0156	1	1	0.0004	0.1417
neuro.soi^b^	1	1	0.7300	1	1	1
alt/ast > 1000.soi	1	1	1	1	1	1
inpatient	0.6296	1.5e-06	7.6e-05	0.0003	< 2e-16	1.6e-14

+Warning Sign, ^a^Severe Plasma Leakage, ^b^Severe Organ Involvement, ^c^Chi-Square test: H0 = all the proportions are equal; H1 = At least one proportion is different. ^c^Based on Holm [25]

The variables rash, tourniquet positive test, narrow pulse pressure, cold skin, impaired consciousness and AST or ALT> = 1000 did not have any influence on the cluster’s division (*p*-values > 0·05). Alternatively, the variables nausea/vomit and persistent vomiting differentiated all clusters (*p*-values < 0·001). These variables were also more frequent in children ≤18 years old than in adults (Table 2).

The patient’s status (outpatient/inpatient) had also showed to be significant in the cluster’s division except for clusters 1 and 3 that disclosed no difference in the distribution of this variable (*p*-value: 0·6296).

The variables history of abdominal pain, leukopenia, and warning signs such as abdominal pain or tenderness, clinical fluid accumulation, mucosal bleeding, lethargy, restlessness, liver enlargement and increase hematocrit associated with a decrease in platelets count were responsible for the cluster 4 definition. The variables that define shock (respiratory distress, rapid and weak pulse and slow capillary filling) were also more significant in distinguishing cluster 4 from the others. The only exception was the variable cold clammy skin/cyanosis that did not show any difference between the clusters. Other variables such as edema and hypotension were responsible only for distinguishing clusters 1 and 3 from cluster 4 but did not show any difference between clusters 1, 2 and 3.

Whereas mucosal bleeding distinguished clusters 1 and 3 from 2 and 4, it also differentiated clusters 2 and 4. Cluster 2 shared characteristics with cluster 4 (petechiae) and clusters 1 and 3 (myalgia, arthralgia and pain behind the eyes) which define this cluster as an intermediary profile. Dehydration was the only variable that differentiated cluster 1 from the others including cluster 3 which shares similar characteristics with cluster 1.

## Discussion

Combined unsupervised machine learning methodologies were useful to identify natural patterns in clinical dengue data, leading to the identification of four well defined clusters profiles. Two clusters (1 and 3) had more than 70% of the patients classified as Dengue without Warning Signs only (*p-value*: 5·11e-14), which could be denominated as low-risk patients. Cluster 4 had the highest percentage of patients classified as severe dengue (*p-value:*5·55e-09) which could be labeled as high-risk group (Table 4). By using similar methodology, Faisal et al. [10] found five natural clusters that could also be clustered in two major clusters as lower risk and higher risk. However, these authors considered only laboratory data (numerical data) of patients in critical phase whereas here we included continuous and categorical variables characterized by the WHO guideline [2] to classify dengue patients in all phases (acute, critical and recovery).

The analysis of all phases of the disease suggests that, besides the rapid evolution of dengue, the transition from dengue without warning signs to dengue with warning and severe dengue happens gradually and it may be linked to the age of the patients. Patients classified as dengue without warning signs (Cluster 1) showed a higher percentage of patients in the acute phase (up to 3 days after the onset of symptoms) whereas cluster 4, with the highest percentage of severity, had a higher percentage of patients between the critical and recovery phases (5–7 days). The same was not observed for clusters 2 and 3. These clusters grouped the patients in the end of the acute phase (Cluster 3) and in the beginning of the critical phase (Cluster 2) (Fig. 4). Nevertheless, these results should be further explored in future studies including age-dependence of infection and clinical presentation in a longitudinal approach.

Age appeared as the most remarkable variable in the cluster’s division showing statistical significance between clusters (Fig. 5). While the low-risk cluster 1 showed a higher concentration of older patients (40–80 years old), the high-risk cluster 4 was characterized mostly by children (5–15 years old). This can be explained by dengue’s age shifting in 2007 in Brazil, when there was an increase of 53% of severe cases occurring in children under 15 years old, and the association of occurrence of severe dengue and hospitalization in younger patients [26]. By simulating the force of infection of dengue based on an age stratified seroprevalence dataset, Rodriguez-Barraquer et al. [27] proposed that the conditions for the age shifting in Brazil were being set gradually and that they represent the transition from re-emergence to hyperendemicity. Our study suggests a similar association because more than 60% of patients in the high-risk cluster were younger than 10 years old (Fig. 5), so that age must be the first characteristic to be considered in dengue hyperendemic areas such as Brazil. The high-risk cluster was mainly characterized by young inpatients showing signs or symptoms of shock (Table 5), justifying the high rate of hospitalization in this cluster. Warning signs such as clinical fluid accumulation, abdominal pain, leukopenia, mucosal bleeding, lethargy, restlessness, liver enlargement and increase hematocrit (hemoconcentration) associated with the decrease of platelets (thrombocytopenia) were also crucial to discriminate this cluster from the others (Table 5). In our exploratory analysis we also found some of these symptoms more frequent in children ≤18 years old than in adults (Table 2). Wakimoto et al. [28] confirmed that abdominal pain, bleeding, lethargy, liver enlargement, hemoconcentration with thrombocytopenia were independently associated with severe dengue in children. Our results confirm these findings and the need for monitoring these parameters in children with dengue.

There was divergent clinical presentation among the low-risk clusters. Besides these clusters sharing a higher frequency of Dengue without warning signs symptoms such as arthralgia, myalgia, and retroorbital pain, the variable dehydration was the only variable discriminating cluster 1 from the others (Table 5). In a prospective observational study in adults with median 35 years old, Thomas et al. [29] observed that dehydration and electrolyte loss was associated with severe patients with symptoms of presyncope, intense weakness, prolonged gastrointestinal symptoms, hypotension and no evidence of plasma leakage. Indeed, cluster 1 presented the lowest percentage of patients with dehydration and highest percentage of patients classified as Dengue without warning signs, indicating a good prognosis.

Cluster 2 showed intermediary characteristics, holding the second highest percentage of patients with warning signs and severe dengue but also sharing characteristics with the low-risk clusters (myalgia, arthralgia and pain behind the eyes) (Table 5). Kuo et al. [30] showed that elderly patients with dengue had significantly higher frequencies of vomiting, mucosal bleeding; higher WBC count, AST and ALT levels, and lower platelet count; when compared with their younger counterparts in critical phase. As cluster 2 was characterized by patients varying in age including children and the elderly, this cluster characteristics could represent the extreme age group of patients presenting warning signs. However, the higher percentage of children with warning signs and the lower number of elderlies included in this study makes this assumption not conclusive. Further studies are needed to characterize differences between the clinical profile in these ages.

The major limitation of this study was its cross-sectional design with retrospective data collection. Although patients were prospectively followed, clinical manifestations could have been incompletely recorded, especially among less severe cases. Therefore, the variables tourniquet positive test, narrow pulse pressure, impaired consciousness and AST/ALT levels that showed to not have any influence on the division of the clusters, need to be evaluated in more detail since the frequency of these variables were lower when compared with the others. Alternatively, the diversity of clinical profiles included in this study (ambulatory/hospitalized patients, adults/children) was an advantage, as it allowed visualization of the categorization of the full spectrum of dengue clinical manifestations. The co-circulation of several arboviruses in an endemic area such as Rio de Janeiro is a drawback that should also be considered. This study had the advantage of being conducted before the emergence of Zika and Chikungunya in the country, as they can present similar clinical manifestations.

Lastly, besides the descriptive design, this study was able to identify natural patterns in dengue clinical profile, giving insights of which clinical factors should be carefully considered in a hyperendemic area.

## Conclusions

Dengue has a wide profile of clinical manifestations and the complexity of the cases with many overlapping levels of its severity has created many difficulties for the physician to predict the disease progression. Our study showed that computational techniques can be useful to identify patterns in the clinical profile of patients with dengue. Our findings suggest that age must be the first characteristic to be considered. Warning signs such as abdominal pain or tenderness, clinical fluid accumulation, mucosal bleeding, lethargy, restlessness, liver enlargement and increase hematocrit should be closely monitored, mainly in children. Further studies exploring these results in a longitudinal approach could be useful to create models to help clinicians and pediatricians to predict severity in dengue infection, mainly in areas where others arbovirus also circulates.

## Data Availability

The datasets generated and/or analyzed during the current study are not publicly available due sensitivity of the data, but the scripts used for the analysis are available from the corresponding author on reasonable request.

## Abbreviations

abpain.ws: Abdominal pain or tenderness
abpainhist: History of abdominal pain
alt/ast > 1000.soi: Aspartate transaminase/alanine transaminase > = 1000
athral: Arthralgia
bleed.ws: Mucosal bleed
BMU: Best Matching Unit
cfa.ws: Clinical fluid accumulation (pleural effusion/ascites)
coldskin.spl: Cold clammy skin/cyanosis
dehyd.spl: Dehydration
DENV: Dengue Virus
edema.spl: Edema
hto/plt.ws: Increase hematocrit + decrease of platelet count
hypo.spl: Hypotension
IgM: Immunoglobulin M
letha/rest.ws: Lethargy, restlessness
leukop: Leukopenia
liveren.ws: Liver enlargement > 2 cm
myal: Myalgia
nau/vom: Nausea/ vomiting
neuro.soi: Impaired consciousness
NS1: Non-structural protein 1
pet: Petechiae
PP: Pulse pressure
pp. < 20 mmHg.spl: Narrow pulse pressure < 20 mmHg,
pvom.ws: Persistent vomiting
rash: Rash
respdist.spl: Respiratory distress
retpain: Pain behind the eyes (retro-orbital eye pain)
RT-PCR: Reverse transcription polymerase chain reaction
rwp.spl: Rapid and weak pulse
scf.spl: Slow capillary filling
severeb: Severe bleeding
SOI: Severe organ involvement
SOM: Self-organizing maps
SPL: Severe Plasma Leakage
tourniq+: + tourniquet test
WHO: World Health Organization
WS: Warning signs
